# Taurine as a key intermediate for host-symbiont interaction in the tropical sponge *Ianthella basta*

**DOI:** 10.1038/s41396-023-01420-1

**Published:** 2023-05-15

**Authors:** Florian U. Moeller, Craig W. Herbold, Arno Schintlmeister, Maria Mooshammer, Cherie Motti, Bettina Glasl, Katharina Kitzinger, Faris Behnam, Margarete Watzka, Thomas Schweder, Mads Albertsen, Andreas Richter, Nicole S. Webster, Michael Wagner

**Affiliations:** 1 Centre for Microbiology and Environmental Systems Science, Division of Microbial Ecology, University of Vienna, Vienna, Austria; 2 Large-Instrument Facility for Environmental and Isotope Mass Spectrometry, Centre for Microbiology and Environmental Systems Science, University of Vienna, Vienna, Austria; 3 Australian Institute of Marine Science, Townsville, QLD Australia; 4 Centre for Microbiology and Environmental Systems Science, Division of Terrestrial Ecosystem Research, University of Vienna, Vienna, Austria; 5 Institute of Marine Biotechnology e.V., Greifswald, Germany; 6 Institute of Pharmacy, Pharmaceutical Biotechnology, University of Greifswald, Greifswald, Germany; 7 Center for Microbial Communities, Department of Chemistry and Bioscience, Aalborg University, Aalborg, Denmark; 8 Australian Centre for Ecogenomics, School of Chemistry and Molecular Biosciences, University of Queensland, St Lucia, St Lucia, QLD Australia; 9 Australian Antarctic Division, Kingston, TAS Australia

**Keywords:** Environmental microbiology, Microbial ecology, Stable isotope analysis, Proteomics

## Abstract

Marine sponges are critical components of marine benthic fauna assemblages, where their filter-feeding and reef-building capabilities provide bentho-pelagic coupling and crucial habitat. As potentially the oldest representation of a metazoan-microbe symbiosis, they also harbor dense, diverse, and species-specific communities of microbes, which are increasingly recognized for their contributions to dissolved organic matter (DOM) processing. Recent omics-based studies of marine sponge microbiomes have proposed numerous pathways of dissolved metabolite exchange between the host and symbionts within the context of the surrounding environment, but few studies have sought to experimentally interrogate these pathways. By using a combination of metaproteogenomics and laboratory incubations coupled with isotope-based functional assays, we showed that the dominant gammaproteobacterial symbiont, ‘*Candidatus* Taurinisymbion ianthellae’, residing in the marine sponge, *Ianthella basta*, expresses a pathway for the import and dissimilation of taurine, a ubiquitously occurring sulfonate metabolite in marine sponges. ‘*Candidatus* Taurinisymbion ianthellae’ incorporates taurine-derived carbon and nitrogen while, at the same time, oxidizing the dissimilated sulfite into sulfate for export. Furthermore, we found that taurine-derived ammonia is exported by the symbiont for immediate oxidation by the dominant ammonia-oxidizing thaumarchaeal symbiont, ‘*Candidatus* Nitrosospongia ianthellae’. Metaproteogenomic analyses also suggest that ‘*Candidatus* Taurinisymbion ianthellae’ imports DMSP and possesses both pathways for DMSP demethylation and cleavage, enabling it to use this compound as a carbon and sulfur source for biomass, as well as for energy conservation. These results highlight the important role of biogenic sulfur compounds in the interplay between *Ianthella basta* and its microbial symbionts.

## Introduction

Marine sponges are important components of coral reefs with a range of critical ecological functions [1]. Sponges play a crucial role in maintaining biomass in reef ecosystems by filtering dissolved organic matter (DOM) from large quantities of water and capturing it as biomass, which is then shed as particulate organic matter (POM) and passed to higher trophic levels [2–4]. Marine sponges often harbor dense, diverse, and stable communities of microorganisms that are thought to play a role in DOM processing, as well as a broad suite of other nutrient transformations [3, 5, 6]. These microbial symbionts have been shown to photosynthetically fix carbon [7], fix N_2_ [8], nitrify [9, 10], as well as carry out anammox, denitrification, and sulfate respiration [11, 12]. Furthermore, isotope labeling experiments have proven that symbiont-derived carbon and nitrogen can be found in sponge cells [7, 11, 13, 14], with more recent studies revealing an emerging view that not only microbial symbionts can incorporate DOM, but that sponge cells do as well, and can even translocate DOM-derived carbon and nitrogen to their symbionts [15–17].

Whilst the specificity, biogeography, and environmental sensitivity of the relationship between sponges and their microbiome has been intensively studied [18–20], our understanding of the physiological interactions between sponges and their symbiotic microorganisms is still very limited. Genome-based investigations of the sponge microbiome have revealed an array of diverse metabolic potential encoded in symbiont genomes, including the processing of organic carbon, nitrogen, and sulfur compounds [21–24], autotrophic sulfur oxidation [25, 26], and vitamin synthesis [27, 28]. These metabolic processes may be mediated by a diverse array of transporters, encoded in the symbionts, to facilitate exchange with the host, as well as the surrounding environment (reviewed in [29]). In a few studies, metaproteomic analyses have revealed the expression of proteins indicative of metabolic interactions between the host and symbionts, including transport functions for typical sponge metabolites, and the potential mediation of host-symbiont interactions via the expression of eukaryotic-like proteins and cell-cell mediators [10, 30]. Despite these insights obtained from a variety of approaches, the high complexity of sponge holobionts, coupled with the inherent difficulty in culturing sponge symbionts, has made it challenging to irrevocably link host interactions and symbiont physiology with symbiont phylogeny.

One way of examining host-symbiont interaction, is by tracking compounds that may be translocated from the host to the symbiont, such as taurine. Taurine has been reported from a broad suite of sponge species, oftentimes as one of the most abundant free amino acids (up to 39% of the free amino acid pool; [31]). Free-living marine bacteria can utilize taurine produced by phytoplankton [32–34] and the genetic capacity for taurine utilization has been identified in many marine sponge microbiomes [25, 26, 35–37]. Taurine has been proposed to be an important intermediate driving metabolic exchange between marine bacteria and eukaryotic phytoplankton [32–34, 38], and yet, to date, there is no direct experimental evidence for the utilization of host-derived taurine by marine symbionts, and of its role in mediating holobiont function, despite the abundance and widespread distribution of this compound in marine animals. However, taurine has recently been demonstrated to confer resistance to pathogens in mammalian gut systems, wherein taurine-metabolizing gut microbiota shield the host against infections, by converting taurine to sulfide, an inhibitor of cellular respiration [39, 40]. Taurine is an ideal osmoregulator, as it is transported by a Na^+^-dependent transport system unique to β-amino acids and so, it is responsive to ionic activities and other osmotic agents [41, 42]. As a zwitterionic compound, extremely high intra- to extracellular concentration gradients for taurine can be maintained, and due to its lipophobic properties, it is not readily lost by diffusion [43]. Taurine also likely functions as a storage and transport compound, providing nitrogen and sulfur to symbionts in the gutless clams *Solemya reidi* [44] and *Solemya velum* [45]. The synthesis of sulfur-containing free amino acids has long been proposed as a strategy for sulfide detoxification and transport in chemoautotrophic symbioses [46–50] and taurine is the dominant free amino acid in many of these species [44, 46–50].

*Ianthella basta* [51] is a widely distributed sponge species found throughout the Indo-Pacific region [52]. In contrast to many marine sponge species with high microbial abundance of microbial symbionts [53], *I. basta* hosts only a low diversity of microbial symbionts and is populated by three dominant phylotypes including an alphaproteobacterium, a gammaproteobacterium and a thaumarchaeote, which all occur at relatively high abundances in the sponge mesohyl [10, 54, 55]. This “low complexity” community is highly stable across different host morphotypes [56], across the large geographic range of the host, under different host health states [55], and across a wide suite of environmental conditions [57]. Thus, the *I. basta* holobiont represents a comparatively tractable model system for the investigation of sponge-symbiont physiological interactions. Recently, we were able to demonstrate that the thaumarchaeal symbiont of *I. basta* —’*Candidatus* Nitrosospongia ianthellae’ — is an active ammonia oxidizer and that, surprisingly, this sponge does not harbor a bacterial nitrite oxidizer [10]. However, the physiology of the remaining two symbiont phylotypes remains unknown. In this study, we elucidate selected physiological traits of the gammaproteobacterial *I. basta* symbiont, which we named ’Candidatus Taurisymbion ianthellae’. Based on annotation of the metagenome-assembled genome (MAG) of this symbiont, we focused on the important biogenic sulfonate intermediate, taurine. We document the expression of a previously described [58], but infrequently observed pathway for the transport and dissimilation of taurine into sulfite, followed by sulfite oxidation into sulfate, encoded by the symbiont, and provide experimental physiological support for this sponge-microbe interaction. For this purpose, we performed whole sponge incubations with added taurine and detected the predicted secreted end product, sulfate. We also employed the use of stable isotope-labeled taurine to track the incorporation of taurine-derived carbon and nitrogen into *I. basta* holobiont biomass, as well as the dissimilation of the nitrogen moiety to ammonium and its subsequent oxidation to nitrite.

## Materials and methods

### Sponge collection for metaproteogenomics and *Ianthella basta* holobiont taurine incubations

Two specimens of the purple thick morphotype of the sponge *Ianthella basta* were collected in waters offshore from Orpheus Island (18°36.878’S, 146°29.990’E), Queensland, Australia, in October 2010 and 2011, and processed as described in [10] for metaproteogenomic analysis. In addition, sponge explants were used for 48-h batch incubations, either in the presence or absence of taurine. The sponge explants used for the taurine incubations were derived from two large yellow thin morph *Ianthella basta* individuals (approx. 0.5 m × 0.5 m × 0.5 cm) collected from Davies Reef (18°49.354 S, 147°38.253 E) on 21st November 2014 and 25th August 2015. Upon collection, the individuals were maintained in flow-through reef seawater onboard the RV Cape Ferguson until their return to the Australian Institute of Marine Science (AIMS) where the individuals were cut into small sponge explants between 10 × 10 × 0.5 cm and 5 × 5 × 0.5 cm and left to heal in aquaria overnight. Since *I. basta* individuals are thin, erect, and laminar, while also exhibiting incurrent (ostia) and excurrent holes (oscula) that are spaced approximately 1 cm apart and on opposing sides of the sponge, relatively small samples can be representative of the entire aquiferous system [59]. The explants were prepared by cutting rectangular-like shapes, while preserving both sides of the fan-shaped sponge, thereby retaining multiple oscula and ostia on both sides. All explants were monitored at the beginning and end of the incubations for tissue health, and no major signs of tissue health regression or necrosis were observed. Furthermore, the edges of the cut sides are estimated to represent 4–8% of the volume of each explant (by assuming an edge thickness of 0.1 cm) and thus, the area of tissue wounds are small relative to the size of each explant. Live sponge explants were transferred to sulfate-free artificial seawater (SFASW; see Supplementary Note for media details) and rinsed three times to remove as much sulfate from seawater as possible. For all incubation experiments, *I. basta* explants were incubated at 25 °C in the dark, in continuously stirred acid-washed glass tanks, either filled with 0.2 µm natural filtered seawater (FSW) or SFASW, and with or without taurine added at different time points and concentrations. All incubations were conducted in the dark to preclude light inhibition of nitrification. SFASW incubations were conducted to more readily discern the production of sulfate from the taurine-dissimilation/sulfite oxidation pathway, since natural seawater contains sulfate at high concentrations. In the first set of experiments, 1 mM unlabeled taurine was added at the beginning of the incubations and another 0.6 mM pulse was added at t = 36 h. For stable isotope incubations in standard seawater, ^15^N and ^13^C labeled taurine (2-Amino-^15^N-ethanesulfonic acid: Taurine-^15^N, 98 atom%; 2-Aminoethane-^13^C2-sulfonic Acid: Taurine-^13^C2, 99 atom%; Campro Scientific GmbH, Berlin, Germany) were added in equimolar concentrations (i.e., a total of 0.8 mM of ^15^N-taurine and 0.8 mM of ^13^C-taurine were added by the end of the incubations) simultaneously, so as to monitor potential incorporation into *I. basta* holobiont biomass, as well as to track dissimilation into NH_4_^+^, followed by successive oxidation to NO_2_^−^ by the dominant thaumarchaeal symbiont [10]. For these two sets of experiments, incubations were conducted in closed 1 l acid-washed glass aquaria at room temperature and water sampling occurred at t = 0, 12, and 48 h, while sampling of *I. basta* biological material occurred at the end of the experiment at t = 48 h. These two experiments utilized 12 explants (approx. 10 × 10 × 0.5 cm; 9.14 ± 2.4 g sponge wet weight) from the individual collected in November 2014 (3 replicates per treatment with *I. basta* explants: SFASW + *I. basta*; SFASW + taurine + *I. basta*; FSW + *I. basta*; FSW + ^13^C^15^N-labeled taurine + *I. basta*). An additional follow-up experiment was conducted with explants derived from the individual collected in October 2015, in SFASW in closed 250 ml acid-washed glass aquaria but with higher temporal resolution (t = 0, 12, 24, 36, and 48 h), and 1 mM or 100 µM isotopically unlabeled taurine added at the beginning of the incubation. This set of experiments utilized 45 explants (approx. 5 × 5 × 0.5 cm; 2.98 ± 0.82 g sponge wet wt), as each explant was sacrificially sampled at the end of each time point. For all sets of incubations, parallel control incubations were performed under identical conditions, but without *I. basta* explants. All incubations were conducted in triplicates. We treated the explants from the same *I. basta* individual as non-technical replicates, since differences in symbiont distribution within the relatively large sponge individuals used here, may occur.

For chemical analyses, seawater samples (10 ml) were taken from the containers (see above-described set of experiments) and filtered using 0.45 μm Sartorius Minisart cellulose acetate filters (Göttingen, Germany). Duplicate samples for dissolved inorganic nitrogen (NH_4_^+^, NO_2_^−^, NO_3_^−^) were measured on a Seal AA3 segmented flow analyser and referenced against OSIL standards and in‐house reference samples. Sulfate was measured via ion chromatography, followed by detection via suppressed conductivity. Confirmation of taurine uptake by *I. basta* was established following an adapted two-step pre-column fluorometric derivatization HPLC method [60]. *O*-phthalaldehyde-ethanethiol (OPA-ET; Sigma Aldrich; 750 μl), prepared daily in methanol (MeOH; Sharlau), buffered with 0.8 M borate (Sigma Aldrich; pH 11), and aged at 4 °C for 2 h, was added to 100 µl of seawater sample diluted in 900 µl MilliQ H_2_O. After 1 min, 50 μl of fluorenylmethyloxycarbonyl chloride (FMOC-Cl) [Sigma Aldrich], prepared in acetonitrile [Sharlau], and aged for at least 12 h, was added; the higher concentration of the derivatizing reagents was required to ensure complete reaction of the taurine (~mM). After a further 1 min, 10 μl of the derivatized sample was injected onto an Agilent 1100 HPLC system (comprising a binary pump, autosampler, column oven, and fluorescence detector, and operated using Chemstation for LC 3D Rev. A.10.01 and 735 Sampler Software v6.10). Samples (*n* = 2 samples per treatment per timepoint; *n* = 5 replicate injections per sample) were chromatographed on a 250 × 4.6 mm i.d., 5 μm particle size, HILIC (Phenomenex) column under the following conditions: 1 ml min^−1^ flow rate; column maintained at 40 °C; mobile phases comprising MeOH-sodium acetate (0.36 M; pH 7.0)-H_2_O (55:8:37, v/v/v) (A) and acetonitrile-sodium acetate (0.36 M; pH 7.0)-H_2_O (10:5:85, v/v/v) (B); gradient 15% A at 0–2 min, 15–20% A at 2–6 min, 40% A at 6–9 min, 85% A at 9–17 min then held until 20 min; the column was flushed with 100% A at 20–21 min and held for a further 4 min before re-equilibration at 15% A for 5 min; fluorescence detection (excitation: 330 nm, emission: 460 nm). For calibration, a taurine stock solution (1 M; > 99.9% Sigma Aldrich) was prepared in Milli-Q water and a dilution series prepared (10 mM–1 nM). γ-Aminobutyric acid (5 M GABA; Sigma Aldrich) was used as the internal standard. The dilution series was stored at 4 °C. The calibration curve (*R*^2^ = 0.99) was used to quantify taurine in seawater. ^15^N-analyses of ammonium and nitrite from the incubation experiments with stable-isotope labeled taurine are described below.

### Metagenome assembly and genome binning

Sequencing libraries were prepared from the same DNA extracted from the individual collected in October 2010 [10] using the Nextera kit (Illumina) according to the manufacturer’s instructions and concentrations measured using the QuantIT kit (Molecular Probes, Life Technologies, Naerum, Denmark). The libraries were paired-end (2 × 150 bp) sequenced on an HiSeq 2000 System (Illumina) using the TruSeq PE Cluster Kit v3-cBot-HS and TruSeq SBS kit v.3-HS sequencing kit, and on an Illumina MiSeq using v3 sequencing kits (2 × 300 bp). Metagenome reads in fastq format, obtained from the sequencing runs, were end-trimmed at a minimum phred score of 15, a minimum length of 50 bp, allowing no ambiguous nucleotides, and sequencing adaptors removed. Trimmed reads from each dataset were assembled using SPAdes version 3.11.0 [61], using default parameters, and genomes were binned using Metabat v 2.12.0 [62]. The choice of SPAdes was made as previous analyses [10] showed that SPAdes returned higher quality MAGs on average for both the archaeal and gammaproteobacterial symbionts. MAGs from multiple Illumina datasets were dereplicated using dRep [63] and provisionally classified using CheckM [64]. A single high-quality gammaproteobacterial MAG was recovered after dereplication and uploaded to MaGe [65] for annotation. The mapped read pairs (at >98% identity) for the assembly of this MAG represented 3.02% of total QC paired reads and read coverage was approximately 26X.

Two additional MAGs were assembled and binned from paired end reads from SRA runs derived from two sponge species, belonging to the same family as *Ianthella basta* (*Hexadella dedritifera* and *Hexadella* cf. *dedritifera*) [66]. Paired end reads for SRA runs SRR8088660, SRR8088661, SRR8088662, and SRR8088663 were downloaded from NCBI. BBduk (bbmap v37.61) was used to remove adapters and residual phiX (adapters.fa, phix174_ill.ref.fa.gz, ktrim = r k = 21 mink = 11 hdist = 2), then quality filtered (minlen = 99 qtrim = r trimq = 15). Read sets were denoised using BayesHammer (SPAdes v3.15.3) [61] and merged using bbmerge (bbmap v37.61). Reads were assembled using SPAdes v3.15.3 (-k 21,33,55,77 --only-assemble) and metaSPAdes (-k 21,33,55,77 --only-assemble) resulting in 8 assemblies (2 assemblers, 4 datasets). Each read set was mapped against each assembly using bbmap v37.61 (minid = 0.99 idfilter = 0.99 ambiguous = best pairedonly = t killbadpairs = t mappedonly = t) and MAGs were generated using metabat1 and metabat2 algorithms (metabat v2.15). MAGs from each SRA run were then dereplicated with dRep v1.4.3 dereplicate_wf (-comp 40 -con 10 -l 200000) [63]. “Winning” MAGs from each SRA run were combined and dereplicated using dRep v1.4.3 (-comp 40 -con 10 -l 200000 -pa 0.6 --S_algorithm gANI --S_ani 0.965). Reads from each SRA run were remapped to each MAG using bbmap v37.61 (minid = 0.99 idfilter = 0.99 ambiguous = best pairedonly = t killbadpairs = t mappedonly = t) and relative abundance was calculated, by dividing mapped fragments by total fragments, for each metagenomic dataset.

### Cryosectioning and FISH for relative symbiont quantification

The aforementioned *I. basta* individual, collected for metagenomic sequencing in October 2010, was assayed for its microbiological composition using fluorescence in situ hybridization (FISH). Briefly, after sample collection, the *I. basta* specimen was cut into tissue strips of approximately 4 cm^3^, fixed in 4% paraformaldehyde (PFA) for 1 h at room temperature, and stored in ethyl alcohol (EtOH)-phosphate-buffered saline (PBS) (1:1) at −20 °C. For FISH, PFA-fixed samples of *I. basta* were embedded in Neg-50 (Richard-Allan Scientific), a cryogenic water-soluble sectioning medium, and cut to 5-μm thin sections (Leica CM3050 S). We designed specific 16 S rRNA-targeted FISH probes for the visualization of ‘*Ca*. Taurinisymbion ianthellae’ (GamD1137, 5’-CTC AAA GTC CCC GCC ATT-3’) and the dominant alphaproteobacterial symbiont (AlfD729, 5‘-CGGACCTGGCGGCCGCTT-3’). To calculate the relative abundance of ‘*Ca*. Taurinisymbion ianthellae’, we applied the double-labeled FISH probe GamD1137 (Cy3), along with the double-labeled FISH probe for the alphaproteobacterial symbiont (AlfD729 in Fluos), and the double-labeled probe mix EUB338-I, EUB338-II, and EUB338-III (all in Cy5; [67]) in equimolar concentrations. *I. basta* sponge section hybridizations were carried out in the presence of 25% formamide in the hybridization buffer (after formamide concentration optimization), and the stringency of the washing buffer was adjusted accordingly [68]. As a negative control, the nonsense probe nonEUB338-I (reverse complementary probe to EUB338-I; double labeled in FLUOS, Cy3, and Cy5) was applied on one section per well per slide hybridized [69]. Probe-conferred visualizations of fluorescent cells were carried out by a confocal laser scanning microscope (CLSM) (LSM 510 Meta; Zeiss, Oberkochen, Germany). Specifically, labeled bacterial cells were counted by eye in 10 randomly selected fields of view (each field contained an average of 260.6 ± 73 SD cells of ‘*Ca*. Taurinisymbion ianthellae’ at 630X magnification with an optical section thickness of 1 µm) on a 146.25 × 146.25 µm ocular grid. Based on these parameters and measurements, we were able to estimate the average density of ‘*Ca*. Taurinisymbion ianthellae’ within the *I. basta* mesohyl, as well as the fraction of ‘*Ca*. Taurinisymbion ianthellae’ relative to total bacterial cells stained by the EUB338 probe mix.

### DNA extraction from whole sponge tissue and qPCR for symbiont quantification

Between 80–150 mg of *I. basta* sponge tissue was thawed (if previously frozen at −80 °C), rinsed successively (3x) in 1X calcium- and magnesium-free artificial seawater (CMF-ASW; see Supplementary Note for media details), and immediately ground into a paste with a mortar and pestle in liquid N_2_. After resuspension in TE buffer, DNA was extracted from the suspension using the same methods as described elsewhere [10] for DNA extraction prior to symbiont quantification via quantitative PCR (qPCR). DNA was extracted from the two individuals used for metaproteogenomics (see above) as well as three additional yellow thin morphotype healthy individuals from a previous study examining the persistence of microbial symbionts in diseased and healthy specimens of *I. basta* [55]. qPCR was used to quantify the number of 16S rRNA genes of ‘*Ca*. Taurinisymbion ianthellae’, the thaumarchaeal ammonia-oxidizer ‘*Ca*. Nitrosospongia ianthellae’, and the dominant alphaproteobacterial symbiont using specific primers designed for each symbiont phylotype [10].

### Phylogenetic analyses

Sequences for the ‘*Ca*. Taurinisymbion ianthellae’ 16S rRNA species tree were assembled by merging a subset of closely related sequences representing members of sponge specific clusters [70], along with top BLAST hits from representative gammaproteobacterial sequences, and the 16S rRNA sequences from the most closely related members with sequenced genomes. These sequences were aligned via the SINA aligner and the phylogenetic trees constructed in IQ-Tree, version 1.6.2 [71] using the model TN + F + R6 (after model selection using Model Finder; [72] with 10 000 ultrafast bootstraps (UFBoot, [73]). In addition, 16S rRNA gene sequences related to ‘*Ca*. Taurinisymbion ianthellae’, were identified in short read archive (SRA) datasets using IMNGS (www.imngs.org, [74]) with default parameters. Successfully mapped reads were then aligned to the reference alignment using SINA and placed into the reference tree using the Evolutionary Placement Algorithm (EPA; [75]) implemented in RAxML-HPC 8.2.11 [76]. Phylogenomic reconstruction was based on a concatenated amino-acid alignment of 43 marker genes constructed with CheckM [64] and trees were constructed using the model LG + F + R7 (after model selection using Model Finder; [72]) with 1000 ultrafast bootstraps (UFBoot, [73]).

### Proteomics

Proteome analysis was performed as done previously [10]. In brief, protein extracts were separated by 1D PAGE followed by liquid chromatography-based mass spectrometry (1D-PAGE-LC-MS/MS). MS spectra and MS/MS spectra were acquired from eluting peptides in a LTQ Orbitrap Velos hybrid mass spectrometer (Thermo Fisher Scientific, Waltham, MA, USA) [77, 78], and searched against predicted protein sequence databases composed of the *I. basta* symbiont-enriched metagenome bins and common laboratory contaminants using the Sorcerer SEQUEST (v.27, rev. 11) algorithm. Protein identifications were filtered with Scaffold version 3.5.1 [79]. For protein identification, only peptides identified with high mass accuracy (maximum ± 10 ppm difference between calculated and observed mass) were considered and at least two exclusively unique peptides were required to identify a protein. False-discovery rates (FDRs) were estimated with searches against a target-decoy database [80, 81]. For relative quantitation of proteins, normalized spectral abundance factors (NSAF) were calculated for each sample [82] and averaged for all replicates and samples.

### NMR-, LC-MS-based detection and quantification of taurine in *Ianthella basta*

To establish the presence of taurine in *I. basta*, a section of tissue from a yellow thin morphotype was excised by scalpel, weighed wet, and exhaustively extracted with 90% MeOH in MilliQ water. The MeOH extract was lyophilized overnight (Dynavac Freeze-drier). The dry MeOH extract (~5 mg) was dissolved in 1 ml deuterated methanol (CD_3_OD; Cambridge Isotope Laboratories) for analysis by proton nuclear magnetic resonance (^1^H NMR; Bruker Avance 600 MHz, 5 mm CPTXI inverse ^1^H-^13^C/^15^N Z-gradient cryogenically cooled probe, referenced to CD_3_OD 3.31 ppm, standard 1D zg pulse sequence). Given the crude nature of the extract and the likelihood that the diagnostic signals would be unable to be distinguished from similar chemistry in the extract, 20 mg (dissolved in ~1 ml 50% MeOH) was chromatographed over a pre-equilibrated Strata C18-E 55 μm, 70 Å, 1000 mg solid phase extraction cartridge (C18-SPE; Phenomenex) and eluted with 10 ml of H_2_O, 10% MeOH, 50% MeOH and 100% MeOH. The four fractions (Fractions 1–4) were evaporated under nitrogen stream at 40 °C and lyophilized to dryness. Fractions 1–2 were prepared in 100% deuterated H_2_O (D_2_O; Cambridge Isotope Laboratories; referenced at 4.39 ppm) and Fractions 3–4 in 10% CD_3_OD: 90% CD_3_OD (referenced to 3.31 ppm) and analyzed by ^1^H NMR. ^1^H and COSY NMR spectra of 100 μM taurine standard, prepared in D_2_O, was used to confirm identification of taurine in Fraction 1. Final confirmation was provided by spiking the ^1^H NMR sample with 32 μl and 128 μl 100 μM taurine and measuring the increase via integration of the two triplet signals.

To corroborate the NMR assignment, Fraction 1 was also analyzed by LC-MS. A 10 μl aliquot of the aqueous Fraction 1 was injected onto an Agilent 1100 HPLC system coupled to a Bruker Esquire 3000 Quadrupole Ion Trap LC-MS and eluted from a 150 × 3 mm Luna 5μ NH_2_ (Phenomenex) column under the following conditions: 0.8 ml min^−1^ flow rate; column maintained at 40 °C; mobile phases comprised of H_2_O + 0.1% formic acid (A) and MeOH + 0.1% formic acid (B); isocratic elution at 90% A: 10% B for 10 min. The ionspray voltage was set to −4500 V, declustering potential -500V, curtain gas flow 9 l min^−1^, ion source gas 40 PSI, and spray temperature at 350 °C. Data was acquired over the mass range 70–200 *m/z*; product ion MS/MS data was acquired over 50–200 *m/z* to establish the fragmentation pattern. Retention time as well as total and extracted ion chromatographs (positive mode) were compared against a 100 μM taurine standard prepared in MilliQ.

The concentration of taurine in sponge tissue was measured by quantitative NMR (qNMR) [83]. Briefly, five sponge explant samples were extracted and chromatographed over C18–SPE as described above. ^1^H NMR spectra of Fraction 1 (~16 mg dry extract) from each of the five sponge extracts were acquired in 800 μl D_2_O (δH 4.39) at 298 K, with sweep width 12 ppm (7184 Hz), 90° pulse, 35 s relaxation delay, receiver gain 16, 2 dummy scans, 16 acquisition scans and 64 k data points. The external reference signal was calibrated using a stock solution of 2 mM taurine in D_2_O. The concentration of taurine in the NMR samples was determined by comparing the signal integrals of well resolved non-exchangeable protons (2H) centered at 3.26 ppm in a 0.20 ppm window, with that of the reference signal. Concentration was normalized to sponge wet wt.

### Taurine measurement in sponge tissue “pore water”

Taurine concentration was measured in readily extractable water, referred to as pore water, from sponge tissue samples of three yellow thin *Ianthella basta* individuals from Davies Reef. The pore water samples of freshly collected *I. basta* tissue samples (approx. 1 × 0.5 cm; 0.54 ± 0.08 g sponge wet wt) were obtained using the following two methods: first, tissue samples were placed in 2.5 ml plastic syringe barrels (after removal of the plunger) and pore water was squeezed into 2 ml plastic collection tubes by applying pressure with the syringe plunger. For the second method, fresh tissue samples were placed into 0.5 ml plastic centrifuge tubes with perforated bottoms (5 holes punched with a 23 G needle). The perforated tubes were placed into 2 ml plastic collection tubes, and *I. basta* pore water was collected via centrifugation at 1000 g for 1 min. All collected pore water samples were immediately frozen and stored at −80 °C until further analysis.

For taurine analysis, the pore water samples (*n* = 6) were diluted 1:10 in artificial seawater (per l: 26.4 g NaCl, 6.8 g MgSO_4_*7H_2_O, 5.7 g MgCl_2_*6H_2_O, 1.47 g CaCl_2_*2H_2_O, 0.66 g KCl, 0.2 g KH_2_PO_4_, 0.19 g NaHCO_3_), and the remaining debris in diluted pore water samples was pelleted by centrifugation at 10,000 g for 5 min. Taurine in the clear supernatant of the *I. basta* pore water samples was quantified against a taurine standard (0 to 250 µM) prepared in artificial seawater. Samples and standards were derivatized with the AccQ-Tag Ultra Derivatization Kit (Waters^TM^) and subsequently measured on a UPLC-Orbitrap mass spectroscopy system [84] with modifications to the UPLC settings. In brief, samples were measured using a UPLC Ultimate 3000 system (Thermo Fisher Scientific, Bremen, Germany) linked to an Orbitrap Q Exactive HRMS system (Thermo Fisher Scientific) with an electrospray ionization (H-ESI) source. Chromatographic separation was conducted on a Water AccQ-Tag Ultra C18 column (2.1 × 100 mm, 1.7 µm particles; Milford, MA, USA) with an ACQUITY column in-line guard filter (2.1 mm, 0.2 µm; Milford, MA, USA) at 55 °C column temperature. The mobile phase consisted of 0.001% formic acid in MilliQ (eluent A) and 0.001% formic acid in acetonitrile (eluent B). The following solvent gradient was applied: 0–0.5 min kept constant at 0.1% B, 0.5–2.5 min increased to 5% B, 2.5–8 min increased to 20% B, 8–8.25 min increased to 90% B, 8.25–11 min maintained at 90% B, 11–11.2 min decreased to 0.1% B, and 11.2–4.5 min maintained at 0.1% B for column re-equilibration. The flow rate was set to 0.4 ml min^−1^ and the injection volume was 1 µL. The Orbitrap Q Exactive HRMS system was operated using full-mass scan mode (m/z 150–1000) in the ESI positive mode. The automatic gain control (AGC) target values were set to 3 × 10^6^ and the resolution was set to 70,000. The lock mass correction was set to 214.0896 (contaminant from plasticizer). The spray voltage was 3.5 kV, while the capillary temperature was set to 300 °C. The sheath gas consisted of 35 arbitrary units, and the auxiliary gas of 15 arbitrary units.

### Isotopic analysis of whole sponge tissue, total nucleic acids, and ^15^N in NH_4_^+^ & NO_2_^−^ using EA-IRMS

For the determination of ^13^C- and ^15^N-enrichment within the holobiont, sponge tissue samples were rinsed briefly (3x) in 1X CMF-ASW, freeze-dried, ground to a fine powder in a mortar, and stored dry, prior to analysis. δ^13^C- and δ^15^N-values of sponge tissues were determined by an elemental analyzer (EA 1110, CE Instruments, Milan, Italy) coupled to an isotope ratio mass spectrometer (IRMS; Finnigan MAT Delta^Plus^ IRMS with a Finnigan MAT ConFlo III interface). For determination of ^13^C- and ^15^N-enrichment in total nucleic acids, *I. basta* whole sponge tissue was homogenized using the above procedure employing liquid N_2_, followed by nucleic acid extraction according to the procedures outlined in [85]. The extracted nucleic acids were then dried overnight in an oven, and δ^13^C- and δ^15^N-values were determined via the same procedure as above. For quantification of the amount of ^13^C- and ^15^N-derived taurine that was incorporated into *I. basta* holobiont biomass, the following formulas were used:

$$n_{C,taurine} = \frac{{\left( {a_{13C,{{{{{{{\mathrm{sponge}}}}}}}}} - a_{13C,ctrl}} \right)}}{{\left( {a_{13C,taurine} - a_{13C,ctrl}} \right)}}n_{{{{{{{{\mathrm{C}}}}}}}},total}$$

$$n_{N,taurine} = \frac{{\left( {a_{15N,{{{{{{{\mathrm{sponge}}}}}}}}} - a_{15N,ctrl}} \right)}}{{\left( {a_{15N,taurine} - a_{15N,ctrl}} \right)}}n_{{{{{{{{\mathrm{N}}}}}}}},total}$$

where the displayed symbols, abbreviations and designations refer to: *a* — isotope fraction ^13^C/(^12^C + ^13^C) or ^15^N/(^14^N + ^15^N) given in at%; *n*: number of carbon or nitrogen elemental atoms; *sponge* — sponge material; *ctrl* — natural isotope abundance control; *total* — total amount of carbon or nitrogen in the analyzed sample. The denominator in both equations ($a_{^{15}C,N,taurine} - a_{^{15}C,N,ctrl}$) were calculated via a standard two-pool mixing model [86]. For comparability of the measurement values obtained from distinct incubations, *n* was normalized to the wet weight of the utilized *I. basta* explants.

For ^15^N-analysis of NH_4_^+^ and NO_2_^−^, sampled seawater from the glass tanks with the labeled taurine incubations was filtered through pre-combusted GF/Fs (Whatman International; treated for 4 h at 450˚C), and subsequently through 0.2 µm polycarbonate filters (Sartorius). All samples were immediately frozen at −20 °C for later analysis. Prior to shipment to the University of Vienna, samples were thawed at room temperature and the microbial inhibitor phenylmercuric acetate was added (to a final concentration of 10 µM). Upon arrival in Vienna, the samples were promptly stored at −80 °C. For determination of the ^15^N content in ammonia, NH_4_^+^ was extracted from samples via microdiffusion [10, 87]. Briefly, 100 mg MgO and an acid trap (acidified cellulose filter disc enclosed in a semi‐permeable Teflon membrane) were added to 9 ml of sample and 1.5 ml of 3 M KCl. After 5 days shaking at 35 °C, the acid traps were removed, dried over concentrated sulfuric acid, and analyzed for the ^15^N content by an elemental analyzer (EA 1110, CE Instruments, Milan, Italy) coupled to an isotope ratio mass spectrometer (IRMS; Finnigan MAT Delta^Plus^ IRMS with a Finnigan MAT ConFlo III interface). The nitrogen isotope composition of NO_2_^−^ was determined by a method based on the reduction of NO_2_^−^ to N_2_O by using sodium azide under acidified conditions [88]. Briefly, 1 ml sample or standard was transferred to 12 ml exetainer and 1 ml 1 M HCl was added. After purging the vials with helium to expel air-N_2_O from the sample headspace, 150 µl 1 M sodium azide buffer (in 10% acetic acid solution) was injected and the vials were placed on a shaker at 37 °C for 18 h. The reaction was quenched by injecting 250 µl of 10 M NaOH. Derived N_2_O was analyzed using a purge-and-trap GC/IRMS system (PreCon, GasBench II headspace analyzer, Delta Advantage V IRMS; Thermo Fischer). Similar to the above calculations, the amount of taurine-derived NH_4_^+^ and NO_2_^−^ were calculated as follows:

$$n_{NH_4^ + ,taurine} = \frac{{\left( {a_{15N,NH_4^ + } - a_{15N,ctrl}} \right)}}{{\left( {a_{15N,taurine} - a_{15N,ctrl}} \right)}}n_{NH_4^ + ,total}$$

$$n_{NO_2^ - ,taurine} = \frac{{\left( {a_{15N,NO_2^ - } - a_{15N,ctrl}} \right)}}{{\left( {a_{15N,taurine} - a_{15N,ctrl}} \right)}}n_{NO_2^ - ,total}$$

By taking into account that the isotopically labeled nitrite emerges from oxidation of ammonia obtained from taurine dissimilation, the total amount of NH_4_^+^ released by the holobiont through taurine metabolization was calculated via:

$$n_{NH_4^ + ,released} = \left( {n_{NH_4^ + ,taurine,sponge} - n_{NH_4^ + ,taurine,SW}} \right) \\ + \left( {n_{NO_2^ - ,taurine,sponge} - n_{NO_2^ - ,taurine,SW}} \right)$$

where SW refers to the values determined from control incubations utilizing seawater without sponge explants. For comparability of the measurement values obtained from distinct incubations, *n* was normalized to the wet wt of the utilized *I. basta* explants.

### NanoSIMS analysis of *Ianthella basta*

NanoSIMS measurements were undertaken on two of the three *I. basta* explant samples obtained from the ^13^C^15^N-taurine incubations. Specimens were selected for maximum isotope enrichment, based on results from EA-IRMS bulk analysis (0.62 and 0.55 at% ^15^N; 1.19 and 1.15 at% ^13^C). Briefly, approximately 0.5 cm^3^ sections were cut from *I. basta* samples, which had been fixed in 4% PFA and stored in a 1:1 mixture of PBS and 96% ethanol at −20 °C. The thus obtained specimens were then rinsed successively (3x) in 1X calcium- and magnesium-free artificial seawater (CMF-ASW) and gently dissociated in 1X CMF-ASW with a scalpel, mortar and pestle, and a final step of sonication for two 30 s cycles at 35% power using a sonication probe [Sonopuls HD2200 equipped with probe MS73 (output frequency 20 kHz, power 200 W, amplitude: 310 μm), Bandelin, Germany]. The suspensions were then filtered through 0.45 μm cellulose acetate filters (type Minisart, Sartorius, Göttingen, Germany) to remove a large portion of remaining sponge cells and nuclei (microscopic visualizations showed a lack of sponge nuclei after filtration), centrifuged at 10,844 × *g* for 10 min at 4 °C to pellet cells, and resuspended in 50% EtOH/50% 1X PBS solution. To visualize all DNA-containing cells present in the sample, a 30 µl aliquot of the suspension was spotted onto antimony-doped silicon wafer platelets (7.1 × 7.1 × 0.75 mm; Active Business Company, Germany) and dried at 46 °C, before staining with a 0.1 % (w/v) solution of the DNA-binding dye 4,6-diamidino-2-phenylindole (DAPI) (Sigma, Deisenhofen, Germany) at room temperature for 5 min. Excess DAPI was removed by rinsing with distilled water and the slides were air-dried. Samples were then imaged on an epi-fluorescence laser microdissection microscope (LMD, Leica LMD 7000) using a 40× air objective for pre-selection of suitable NanoSIMS measurement areas, which were subsequently marked utilizing the LMD laser. High resolution imaging of fluorescent cells in marked regions was carried out by a confocal laser scanning microscope (CLSM) (LSM 510 Meta; Zeiss, Oberkochen, Germany). NanoSIMS measurements were performed on a NanoSIMS 50 L (Cameca, Gennevilliers, France) at the Large-Instrument Facility for Environmental and Isotope Mass Spectrometry at the University of Vienna. Further details on NanoSIMS data acquisition and analyses are presented in the Supplementary Information.

## Results and discussion

### A metagenome assembled genome and phylogenetic analyses of the dominant gammaproteobacterial symbiont of *Ianthella basta*

**Fig. 1:**
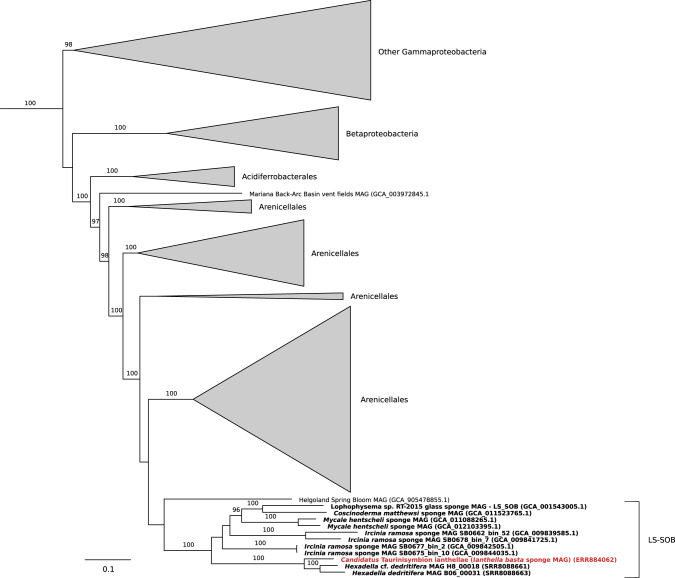
Maximum-likelihood phylogenomic tree of ‘*Ca*. Taurinisymbion ianthellae’ along with other selected proteobacterial symbionts. The phylogenomic tree is based on 43 concatenated universal, single-copy marker genes identified with CheckM, and was generated after automatic model selection with IQ-Tree. ‘*Ca*. Taurinisymbion ianthellae’ is displayed in red bold font while sponge symbionts are displayed in black bold font.

From the extensive metagenomic data set used for genomically characterizing the thaumarchaeal symbiont of *I. basta* [10], we retrieved a 2.35 Mbp MAG consisting of 97 contigs, representing a nearly complete genome (94.8%; possibly originating from several closely related strains) with a very low level of contamination (Table S1). Phylogenetic analysis of the only 16S rRNA gene copy in the MAG demonstrated that this gene specifically clustered at 99% nucleotide identity with previously recovered sequences of the dominant gammaproteobacterial symbionts from *I. basta* (Fig. S1) [10, 55, 56] and fall into the neighborhood of sponge specific clusters 144–8 (SC) [70]. A phylogenomic reconstruction using concatenated single copy conserved marker genes (Fig. 1) [64], confirmed the affiliation of this MAG with the Gammaproteobacteria and revealed that it can be further classified into the Arenicellales order and within the LS-SOB family (originally named after the bacterial MAG extracted from the glass sponge *Lophophysema eversa*, which encodes a sulfur oxidation pathway [89]; based on the Genome Taxonomy Database [90]).

Based on the concatenated gene tree analyses, we further queried representative 16S rRNA gene sequences pulled from LS-SOB genome bins, against the IMNGS 16S rRNA gene amplicon database [74], and placed the hits into the reference 16S rRNA gene tree using the Evolutionary Placement Algorithm [75]. As a result, 3 sets of short reads were placed into the branch leading to the dominant gammaproteobacterial symbiont sequences obtained from *I. basta* (Fig. S1). The closest two groups (I4, I6) comprised 5 (out of 6 total) operational taxonomic unit (OTU) reads derived from unspecified coral and sponge samples, 4 of which were OTU reads from corals sampled from the same study site as *I. basta* for this study (Orpheus Island, Queensland, Australia). 141 OTU reads were placed into the I7 group, of which 29 and 4 were derived from sponge and coral metagenomes, respectively (the other 112 OTU reads were derived from other metagenomic data sets spanning a variety of environmental matrices). Out of this group, three OTU reads were obtained from two sympatric marine sponge species (NE Atlantic) within the same genus, (*Hexadella dedritifera* and *Hexadella* cf. *dedritifera*), both of which are also affiliated with the same sponge family as *I. basta*, the Ianthellidae. These two closely related phylotypes, from *H. dedritifera* and *H*. cf. *detritifera*, respectively, exhibited 16S rRNA gene identities of ~97% (over 418 bp) with each other, 95–97% with the *I. basta* gammaproteobacterial symbiont, and were found to comprise 33–42% of the total amplicon dataset derived from these sponges [91]. Noting that these two Hexadella species harbor abundant and closely related gammaproteobacterial phylotypes, and also contain AOA-symbionts closely related to the AOA in *I. basta* [66], we assembled and binned two new gammaproteobacterial symbiont MAGs from the published raw read data set of these two Hexadella sponges.

The phylogenomic reconstruction confirmed that these two Hexadella gammaproteobacterial symbiont MAGs were most closely related to the *I. basta* gammaproteobacterial symbiont (Fig. 1), and AAI analyses indicated that the *I. basta* gammaproteobacterial symbiont shares AAI values of 76–80% with these two Hexadella gammaproteobacterial symbionts. Among the other LS-SOB genomes retrieved from marine sponges [37, 89, 92–94], the *I. basta* gammaproteobacterial symbiont shares AAI values of 54–59%. Since these identity values signify that the gammaproteobacterial symbiont represents a new species within a new genus along with the other two Hexadella gammaproteobacterial symbionts — 60–80% AAI is typical for organisms grouped at the genus level [95, 96] — we propose the name ‘*Candidatus* Taurinisymbion ianthellae’. Taurinisymbion gen. nov. (Tau.ri.ni.sym’bi.on. N.L. n. taurinum, taurin; Gr. pref. sym, together; Gr. masc. part. n. bion, a living being; N.L. masc. n. Taurinisymbion, a taurin-degrading living partner) for the *I. basta* gammaproteobacterial symbiont. The name *Taurinisymbion* describes this organism’s ability to utilize taurine as a source of energy and biomass whilst residing in symbiosis within a sponge, and we were able to identify the same pathways encoded by ‘*Ca*. Taurinisymbion ianthellae’ for taurine utilization in the two Hexadella gammaproteobacterial symbiont MAGs, thereby suggesting a conserved evolutionary function that may be adaptive for this proposed genus (see below; Table S2). The species name *ianthellae* refers to its discovery and description as a symbiont of the marine sponge, *I. basta*.

### ‘*Candidatus* Taurisymbion ianthellae’ is an abundant symbiont of *Ianthella basta*

**Fig. 2:**
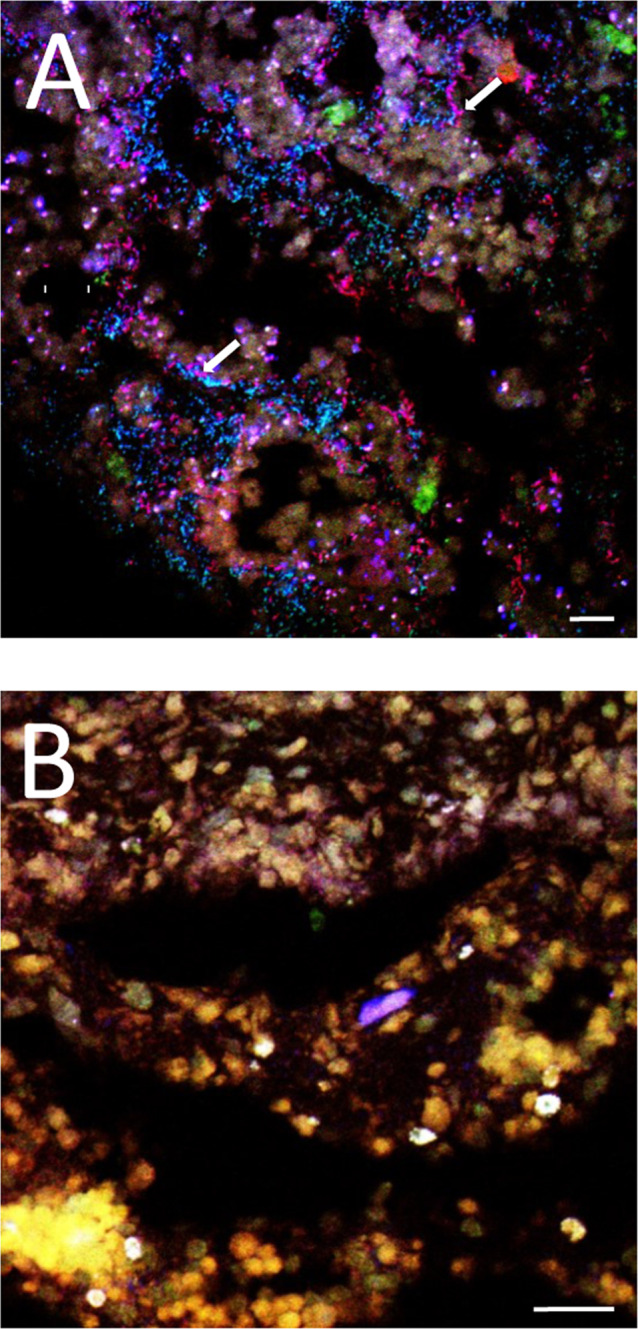
Fluorescence in situ hybridization of 5 μm thick cryo-sections of *Ianthella basta* using double-labeled probe sets. **A** ‘*Ca*. Taurinisymbion ianthellae’ (red), *Alphaproteobacteria* symbiont*-*probe (green), and *Bacteria-*probe (EUB338-I-III; blue); **B** negative controls using the double-labeled non-EUB338 probe set are depicted in all three color channels. The combination of the ‘*Ca*. Taurinisymbion ianthellae’ specific probe signals in red with the general *Bacteria* probe signals in blue, results in the visualization of ‘*Ca*. Taurinisymbion ianthellae’ as magenta signals and representative cells are indicated with white arrows. The alphaprotebacterial symbiont appears in cyan. Green structures represent autofluorescence. Scale bars are 10 µm for reference.

Consistent with the high coverage of reads and peptides assigned to ‘*Ca*. Taurinisymbion ianthellae’ in the metaprotoegenomic data sets from *I. basta* and the dominance of this phylotype in other *I. basta* studies [54–56], we observed a high abundance of this symbiont (Fig. 2) in the mesohyl of *I. basta* using fluorescence in situ hybridization (FISH) with a newly designed specific 16S rRNA-targeted probe. Quantitative FISH revealed that ‘*Ca*. Taurinisymbion ianthellae’ achieved average cell densities of 1.22 ± 0.34 (SD) × 10^10^ cells cm^−3^ sponge and accounted for 24 ± 5.6% (SD) of all cells hybridizing with the probe set EUB338-I-III targeting most bacteria (which does not cover the thaumarchaeal symbiont of *I. basta*). Moreover, absolute quantification of ‘*Ca*. Taurinisymbion ianthellae’ using qPCR with newly designed symbiont specific primers, showed an average absolute abundance of 2.78 ± 1.5 (SD) × 10^10^ 16S rRNA gene copies g^−1^ sponge wet wt, which is in the same range as the population density values observed from the FISH images (assuming that 1.2 g sponge wet wt = 1 cm^−3^; [97]). The absolute abundances obtained for the dominant thaumarchaeal and alphaproteobacterial symbionts are also in the 10^10^ 16S rRNA gene copies g^−1^ sponge wet wt range (Fig. S2). The ratio of ‘*Ca*. Taurinisymbion ianthellae’ 16S rRNA sequences to the total 16S rRNA genes derived from qPCR assays (targeting the gammaproteobacterial-, alphaproteobacterial, and thaumarchaeal symbionts of *I. basta*) was 41.1 ± 34 (SD)%. Nevertheless, it should be noted that qPCR abundances cannot directly be translated to cell numbers as (i) symbionts can have multiple rRNA gene copies (although the ‘*Ca*. Taurinisymbion ianthellae’ MAG has only one 16S rRNA gene copy), (ii) genome polyploidy might occur in sponge symbionts and (iii) extracellular DNA of the symbionts will also be detected. Similarly, using FISH for quantification may result in the undercounting of microbial cells with low ribosome contents.

### ‘*Ca*. Taurinisymbion ianthellae’ expresses proteins for taurine catabolism and subsequent NH_4_^+^ and SO_4_^2^^−^ export

**Fig. 3:**
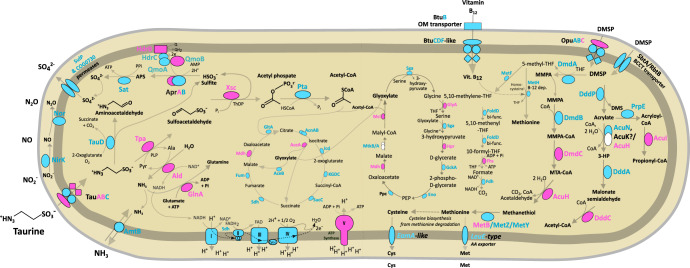
Taurine and DMSP metabolic pathways identified and expressed in ‘*Ca*. Taurinisymbion ianthellae’. Central carbon and nitrogen metabolism in ‘*Ca*. Taurinisymbion ianthellae’ based on metaproteogenomic analyses with a focus on taurine and DMSP metabolism pathways along with the relevant import and export of exchanged metabolites. Depicted are proteins necessary for taurine import, followed by taurine dissimilation, energy conservation via sulfite oxidation and the electron transport chain, along with carbon incorporation via the glyoxylate bypass. Similarly depicted are the proteins necessary for DMSP import as well as the corresponding DMSP cleavage and demethylation pathways, including the oxidation of 5-methyl-THF and its role as a carbon donor for the conversion of glycine to serine. Enzymes detected via metaproteomics are highlighted in fuchsia, while enzymes only identified in the ‘*Ca*. Taurinisymbion ianthellae’ MAG are highlighted in turquoise. Missing pathway genes are depicted as white shapes. AMP adenosine monophosphate, APS adenosine-5’-phosphosulfate, Cys cysteine, DMSP dimethylsulfoniopropionate, DMS dimethylsulfide, 3-HP 3-hydroxypropanoate, Met methionine, MMPA methylmercaptopropionate, MTA-CoA methylthioacryloyl-Coenzyme A, PEP phosphoenolpyruvate, PLP pyridoxal 5’-phosphate, Q quinone, ThDP thiamine diphosphate, THF tetrahydrofolate. Additional information for genes and pathways depicted in the figure can be found in Supplementary Tables S2–S5.

To analyze the metabolic potential of ‘*Ca*. Taurinisymbion ianthellae’, we annotated its MAG by employing the previously presented metaproteogenomic data set from *I. basta* [10] and were able to assign 149 proteins to this symbiont MAG. These proteins represented 35.7%, in NSAF values, of all proteins assigned to the *I. basta* microbiome (Table S3), highlighting the physiological dominance of ‘*Ca*. Taurinisymbion ianthellae’. From this metaproteogenomic dataset, we concluded that ‘*Ca*. Taurinisymbion ianthellae’ is a facultative anaerobe with an ability to metabolize a variety of organic compounds. The genomic potential for the major central metabolic pathways, such as glycolysis, the pentose phosphate pathway, the tricarboxylic acid (TCA) cycle, the glyoxylate bypass, a conventional respiratory chain (complex I–IV; two respiratory complex IV were found to be encoded, a *cbb*_3_- and a *aa*_3_-type cytochrome c oxidase; complex V), and the genes for denitrification of NO_2_^−^ to N_2_O, and DMSP catabolism, were all present in the recovered MAG (Fig. 3, Table S2). A further discussion of a few of these genomic features, with a focus on DMSP metabolism, is provided in the Supplementary Information.

‘*Ca*. Taurinisymbion ianthellae’ encodes a previously described pathway for taurine import and transamination (*tauABC, tpa, xsc*) [98] into sulfite via a desulfonated intermediate sulfoacetaldehyde, as well as a pathway for the subsequent oxidation of the resultant toxic and highly reactive sulfite into sulfate via the cytoplasmic enzymes APS reductase (a*prAB*) and ATP sulfurylase (*sat*) [58]. This pathway consumes AMP and sulfite, and releases ATP via substrate-level phosphorylation and sulfate, while channeling two electrons from the oxidation of sulfite, with its very negative redox potential, to the quinone pool via the membrane-bound QmoAB. Consistent with other sulfur oxidizing bacterial genomes, the *qmoC* gene is replaced by *hdrBC* genes ([99] and references therein), with the *hdrBC* genes found to be adjacent to *qmoAB* in ‘*Ca*. Taurinisymbion ianthellae’, and the HdrB subunit expressed in the holobiont (Fig. 3, Tables S2 and S3). For the potential export of sulfate, two putative sulfate permeases (COG0730 permease family, [100]; COG0659, SulP family permease), were detected in the ‘*Ca*. Taurinisymbion ianthellae’ MAG. The gene encoding taurine dioxygenase (*tauD*), an alpha-ketoglutarate-dependent dioxygenase, which desulfonates taurine into aminoacetaldehyde (the fate of which is currently unknown) and sulfite, was also found within the ‘*Ca*. Taurinisymbion ianthellae’ MAG. TauD is involved in the assimilation of sulfur from taurine in several aerobic bacteria [101]. Furthermore, we found many of these genes (*tauABC*, *tpa*, *tauD*, *xsc*, *aprAB*, *qmoABC*) encoded in the previously mentioned Hexadella gammaproteobacterial symbiont MAGs, which belong to the same proposed genus as ‘*Ca*. Taurinisymbion ianthellae’ (Table S2).

Several of the genes involved in taurine degradation were also found to be expressed after being mapped to the ‘*Ca*. Taurinisymbion ianthellae’ MAG. Specifically, we detected the expression of the taurine transporter TauABC (with TauAB detected only when the 454 metagenome dataset was included as a database for initial metaproteomic analyses), and the two enzymes, taurine-pyruvate aminotransferase (Tpa) and sulfoacetaldehyde acetyltransferase (Xsc), involved in the sequential dissimilation of taurine to sulfite via sulfoacetaldehyde. Additionally, 3 out of 7 proteins necessary for the subsequent oxidation of sulfite to sulfate (AprA, QmoB and HdrB; while Sat was not detected in the metaproteome data set) were expressed. A phosphate acetyltransferase is present in the ‘*Ca*. Taurinisymbion ianthellae’ MAG, which would convert the acetyl phosphate produced from the activity of Xsc (also producing sulfite) into acetyl-CoA, for entry into the TCA and the glyoxylate bypass cycle. We detected the expression of the key enzymes malate dehydrogenase (Mdh) and isocitrate lyase (AceA), from each cycle, respectively, suggesting that the gyoxylate bypass is used for anabolic purposes, while sulfite oxidation is used for energy conservation by ‘*Ca*. Taurinisymbion ianthellae’. In addition to the expressed proteins detected for the processing of taurine-derived sulfur and carbon compounds, we detected the expression of alanine dehydrogenase (Ald), which would replenish the pyruvate needed by the Tpa enzyme for taurine dissimilation (while also providing NADH/H^+^ for respiration) and provide NH_3_ for assimilation into glutamine, by a highly expressed glutamine synthetase (GlnA; 1.30% NSAF). Ammonia can also be transported out of the cell via an encoded ammonium transporter (*amtB*; Fig. 3, Table S2), where it can be readily oxidized to nitrite, as previously demonstrated [10], by the dominant thaumarchaeal symbiont. Excluding the expression of glutamine synthetase, the other detected 6 proteins involved in the catabolism of taurine (Tpa, Xsc, AprA, QmoB, and HdrB) amounted to 0.97% NSAF of the 149 proteins assigned to the ‘*Ca*. Taurinisymbion ianthellae’ MAG. Nevertheless, the detection of the high affinity GlnA suggests that ‘*Ca*. Taurinisymbion ianthellae’ was nitrogen limited at the time that the *I. basta* holobiont individual was sampled in situ. It has been previously observed that the *I. basta* holobiont, along with its thaumarchaeal ammonia-oxidizing symbiont, ‘*Ca*. Nitrosospongia ianthellae’, is nitrogen limited at ambient conditions [10], and this is in line with apparent nitrogen limitation in marine systems [102] and mammalian large intestines [103]. The observations that the *I. basta* holobiont readily dissimilated added taurine in *I. basta* explant incubations (see below) and incorporated the taurine-derived carbon and nitrogen at C:N ratios indicative of nitrogen limitation (see Supplementary Information), further corroborate these findings.

While the expression of the taurine transporter has been observed in the gammaproteobacterial symbiont of *Olavius algarvensis* [104], the recent observations that marine sponge symbionts have the capacity for taurine utilization, is primarily based on gene presence [25, 26, 35–37], with the exception of metaproteomic evidence for *Ca*. Entotheonella phylotypes residing in the marine sponge *Theonella swinhoei*. However, this symbiont expressed multiple encoded copies of TauD-related proteins postulated to possibly function in the breakdown of small aromatic compounds [105]. A recent study by our group performed deep sequencing runs on more *I. basta* individuals — while also conducting metaproteomics — and was able to assemble additional low abundant microbial symbiont MAGs that were previously overlooked [106]. One of these low abundant symbionts is an alphaprotebacterium (Alphaproteobacteria, g_UBA2767) that also possesses the capability to fully convert taurine into ammonium and sulfate via the same pathways as ‘*Ca*. Taurinisymbion ianthellae’. However, this low abundance Alphaproteobacterium only represents 1–5% of the microbial community and did not express any of the proteins involved in taurine import, taurine dissimilation or sulfite oxidation (it only expressed 0.4% of all detected proteins in the metaproteomic dataset). Moreover, this set of metaproteomic analyses was able to detect the TauABC and AprA proteins and definitively assign them to the ‘*Ca*. Taurinisymbion ianthellae’ MAG.

Oxidation of sulfite to sulfate by taurine-degrading bacteria occurs via different pathways. Frequently periplasmic and cytoplasmic sulfite dehydrogenases are used [107, 108], but these enzymes were not detected in the ‘*Ca*. Taurinisymbion ianthellae’ MAG. The genetic capability to carry out the two-step AMP-dependent indirect oxidation of sulfite to sulfate via APS in the cytoplasm employed by this symbiont, is infrequently detected in taurine degraders, with examples found among the ubiquitous, pelagic SAR11 ([58]; but most SAR11 lack ATP sulfurylase), the γ-3 symbiont in the gutless oligochaete, *Olavius algarvensis* [109], and two marine sponge symbionts [25, 26]. Although a metaproteomic study in Antarctic waters has previously revealed the expression of SAR11-associated peptides for this particular pathway of taurine dissimilation (Tpa, Xsc) followed by sulfite oxidation (AprAB) [58], SAR11 in culture has not yet been shown to excrete sulfate in the presence of taurine [110, 111].

### Taurine metabolism in the *Ianthella basta* holobiont

**Fig. 4:**
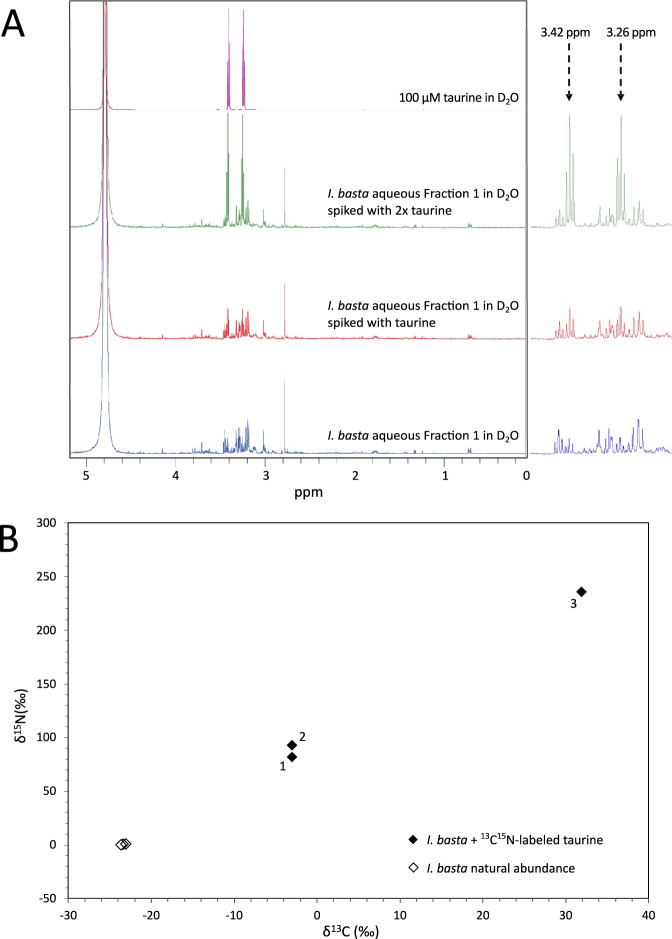
Detection of taurine and incorporation of ^13^C and ^15^N from isotopically labeled taurine in *I. basta* holobiont biomass. **A** Detection of taurine in the aqueous fraction Fraction 1 of the 90% methanol extract of *Ianthella basta* via 1H NMR. ^1^H NMR spectra of Fraction 1 from C18 of crude methanolic extract (blue), Fraction 1 spiked with 32 μl (red) and 128 μl (green) of 100 μM taurine, as well as 100 μM taurine (purple), all acquired in D_2_O. Zoomed regions are depicted of diagnostic triplet signals at 3.26 and 3.42 ppm, and the increase in taurine signal intensity with spiking. **B** Incorporation of ^13^C- and ^15^N-labeled taurine into sponge holobiont biomass at the total nucleic acid level as determined by EA-IRMS. Data from three sponge explants incubated with labeled taurine (numbered for reference) and three control sponge explants incubated with unlabeled taurine are depicted.

Based on the findings of the metaproteogenomic data set, we investigated taurine metabolism in the *I. basta* holobiont experimentally. NMR and LC-MS analyses of the 90% MeOH extract and the aqueous Fraction 1 confirmed that taurine was present in this sponge (Fig. 4A and Fig. S3) [112]. The natural concentration of taurine, determined by qNMR, was 5.48 ± 0.69 µmol g^−1^ sponge wet wt, which is in the mid-range of previously reported values for six other marine sponges (1.05–12.5 μmol g^−1^ sponge wet wt sponge) [113]. Furthermore, taurine measurements in *I. basta* pore water samples ranged between 819 µM and 1352 µM, with no significant difference (t-test, *p* = 0.2754) between the two pore water extraction methods employed. Congruently, we were able to detect ^13^C and ^15^N enrichment in whole sponge and total nucleic acids elemental analyzer isotope ratio mass spectrometry (EA-IRMS) analyses of *I. basta* sponge explants that underwent additions of an equimolar mixture of ^13^C- and ^15^N-labeled taurine in 48 h incubations (Fig. 4B and Fig. S4). While it cannot be excluded that sorption of labeled taurine contributed to the isotopic enrichment in the whole sponge experiments, the detection of labeled total nucleic acids clearly indicated assimilation and incorporation of labeled taurine into *I. basta* holobiont biomass. Thus, from the whole sponge EA-IRMS data, we were able to estimate uptake rates of taurine-derived C and N assimilated into whole *I. basta* holobiont biomass and obtained values of 1.38 ± 0.3 nmol C min^−1^ g^−1^ sponge wet wt (±SE) and 0.74 ± 0.2 nmol N min^−1^ g^−1^ sponge wet wt (± SE). These *I. basta* holobiont taurine-derived carbon uptake rates correspond to approximately 4–10% of the bulk DOC uptake rates reported for the more diverse microbiome of the marine sponge *Aplysina aerophoba* [17], thereby highlighting the importance of taurine in the *I. basta* holobiont. Moreover, NanoSIMS analyses conducted on dissociated sponge extracellular material revealed accumulation of taurine-derived C and N within regions (Figs. S5 and S6) that were similar in size and shape to typical sponge microbial symbiont cells previously visualized in *I. basta* by transmission electron microscopy [55, 114].

### Taurine catabolism by *Ianthella basta* holobiont results in the accumulation of inorganic S and N species

**Fig. 5:**
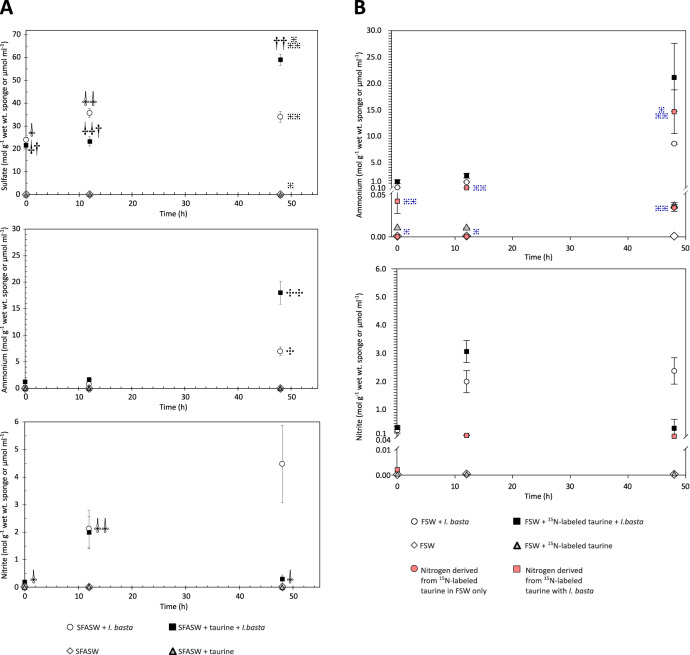
Time series of dissolved sulfate, ammonium, and nitrite concentrations in incubation media of *I. basta* holobiont batch experiments. **A** 48 h incubation in sulfate-free artificial seawater (SFASW) performed either without (white circle) or with added unlabeled taurine (filled square: *t* = 0, +1 mM taurine; t = 36 h, +0.6 mM taurine). Concentrations are normalized to the wet weight of the respective *I. basta* explants applied in the incubations (8.83 ± 2.38 SD g sponge wet wt.; range: 6.5–13.4 g sponge wet wt.). The data from the corresponding control experiments with SFASW without *I. basta* holobiont are displayed as white diamonds (without added taurine) and gray triangles (with added taurine) and plotted on the same axes, given in µmol ml^−1^. A starting average concentration of 0.2 mM sulfate was measured in all incubation treatments containing *I. basta* explants most likely representing residual carryover from the sponge material. Incubations were performed in biological triplicates and water samples from each incubation were taken at each time point. In **A**,  symbols for sulfate concentrations denote significant difference in pairwise comparisons of incubations from t = 0 () to t = 12 () for both the incubations with *I. basta* and taurine and without added taurine (one-tailed *t*-test, *p* < 0.05);  symbols in the sulfate concentration panel reflect significant differences for sulfate concentrations for *I. basta* and taurine incubations at t = 48 () when compared to the earlier time points () (one-way ANOVA followed by Holm-Sidak tests, *p* < 0.001); similarly, the number of ⁜ symbols reflects significant differences for sulfate concentrations for incubations with *I. basta* and taurine when compared to all other treatments at t = 48 h (one-way ANOVA followed by Holm-Sidak tests, *p* < 0.001).  in the ammonium concentration panel reflects a significant difference between incubations with *I. basta* and taurine and only *I. basta* () (two-tailed *t*-test, *p* < 0.05).  in the nitrite concentration panel reflects a significant difference between time points for the incubations with *I. basta* and taurine (one-way ANOVA followed by Tukey pairwise multiple comparison tests, *p* < 0.05). **B** Ammonium and nitrite accumulation in *I. basta* holobiont batch incubations (48 h) in natural filtered seawater (FSW) performed either with added taurine (filled symbols: t = 0, +1 mM taurine; t = 36 h, +0.6 mM taurine) or without added taurine (9.45 ± 2.53 SD g sponge wet wt; range: 5.5–12.7 g sponge wet wt). Taurine was added as an equimolar mixture of ^13^C- and ^15^N-labeled taurine to obtain sponge material for subsequent EA-IRMS and NanoSIMS analyses (by sacrificially sampling sponges at the end of the experiment) as well as to measure ^15^N in NH_4_^+^ and NO_2_^−^. Incubations were performed in biological triplicates and water samples from each incubation were taken at each time point. The data from the corresponding control experiments with FSW and without *I. basta* holobiont explants are displayed as white diamonds and gray triangles as in **A**). Pink symbols represent the measured concentrations of ^15^N in ammonium and nitrite that were a product of the added ^15^N-labeled taurine (t = 0, +0.5 mM ^15^N-labeled taurine; t = 36 h, +0.3 mM ^15^N-labeled taurine). The number of ⁜ symbols in the ammonium concentration panel in **B**) reflects significant differences in ^15^NH_4_^+^ concentrations measured in incubations with *I. basta* and isotopically labeled taurine and in incubations without *I. basta* between different time points and across treatments (one-way ANOVA followed by Student-Newman-Keuls pairwise multiple comparison tests, *p* < 0.05). Error bars refer to + /− 1SE.

Some microbial pure cultures catabolizing taurine, oxidize the dissimilated sulfonate moiety to sulfate, which is subsequently excreted [115–117]. Considering that metaproteogenomic analyses predicted sulfate as a secreted end product of ‘*Ca*. Taurinisymbion ianthellae’ taurine metabolism, we analyzed whether the *I. basta* holobiont produced sulfate (SO_4_^2-^) in experiments with and without added taurine. Specifically, we conducted 48 h incubations with *I. basta* explants amended with taurine (in total 1.6 mM) and without taurine in SFASW. SFASW was used in these incubations to facilitate the detection of sulfate formation by the holobiont. Pairwise comparisons in incubations with sponges in SFASW without the addition of taurine (SFASW + *I. basta*), resulted in a small, but significant production of SO_4_^2-^ after 12 h, indicating in vivo production of SO_4_^2-^ (*p* < 0.05, one-tailed *t*-test) (Fig. 5A). In incubations with *I. basta* explants and added taurine (SFASW + *I. basta* + taurine), we observed a similarly small increase at 12 h (*p* < 0.05, one-tailed *t*-test), with SO_4_^2-^ production strongly increasing between the 12 h and 48 h time points, suggesting a metabolic lag effect (Fig. 5B). Moreover, the final SO_4_^2-^ concentrations of 590 ± 89 (SE) µM SO_4_^2-^ (59.0 ± 2.0 µmol g^−1^ sponge wet wt in Fig. 5A) at 48 h were significantly higher than the SO_4_^2-^ concentrations at t = 0 and t = 12 h (one-way ANOVA followed by Holm–Sidak tests, *p* < 0.001) and also significantly higher than all control incubations, including the incubations with sponge explants but without taurine at t = 48 h (one-way ANOVA followed by Holm-Sidak pairwise multiple comparison tests, *p* < 0.001). Two additional incubation experiments, with refined temporal resolution (t = 0, 12, 24, 36 and 48 h) using 1 mM and 100 µM unlabeled taurine amendments, respectively, resembled the results of the previous experiment, and displayed a significant SO_4_^2-^ increase after 48 h for both taurine treatments (one-way ANOVA followed by Tukey pairwise multiple comparison tests, *p* < 0.05), with essentially a full recovery of the +100 µM taurine sulfonate in the form of SO_4_^2^^−^ after 48 h (Fig. S7).

We observed NH_4_^+^ and NO_2_^−^ production in *I. basta* incubations, with and without added unlabeled taurine (SFASW + *I. basta* + taurine; SFASW + *I. basta*), where NH_4_^+^ production at the end of the experiment with added taurine, drastically exceeded those of *I. basta* incubations without added taurine, consistent with the production of ammonium from taurine (Fig. 5A; *p* < 0.05, two-tailed *t*-test). Furthermore, a clear increase in NO_2_^−^ production occurred within the first 12 h and was found to be significant for incubations with added taurine (Fig. 5A, SFASW + *I. basta* + taurine: one-way ANOVA followed by Tukey pairwise multiple comparison tests, *p* < 0.05). NH_4_^+^ and NO_2_^−^ production in *I. basta* has previously been observed [10] and the NO_2_^−^ production was attributed to the only ammonia-oxidizing symbiont, ‘*Ca*. Nitrosospongia ianthellae’. At the end of the experiment, NO_2_^−^ concentrations decreased in the incubations with added taurine, consistent with the previously demonstrated inhibition of ammonia oxidation at concentrations ≥100 µM NH_4_^+^ in the *I*. basta holobiont and/or with the concomitant increased hypoxia facilitating denitrification activities [10], a capability that is encoded in ‘*Ca*. Taurinisymbion ianthellae’. Additional experiments with refined temporal resolution, displayed the same general trends (Fig. S7).

FSW incubations of the *I. basta* holobiont with ^15^N-labeled taurine were performed. In these experiments, we were able to detect ^15^NH_4_^+^ and ^15^NO_2_^−^ formation by *I. basta* sponge explants (Fig. 5B), demonstrating taurine metabolism and oxidation of taurine-derived ammonia to nitrite by the sponge holobiont. Specifically, this indicates that the dominant thaumarchaeal symbiont hosted by *I. basta*, ‘*Ca*. Nitrosospongia ianthellae’, which was previously shown to be the only resident ammonia-oxidizer in this holobiont [10], oxidized the taurine-derived ammonia to nitrite. The amount of ^15^NH_4_^+^ and ^15^NO_2_^−^ derived from ^15^N-labeled taurine in incubations with *I. basta* was always higher than the amount detected in FSW control incubations without *I. basta* at t = 12 and t = 48 h (Fig. 5B; one-way ANOVA followed by Student–Newman–Keuls pairwise multiple comparison tests, *p* < 0.05; ^15^NO_2_^−^ in FSW control incubations were undetectable in all samples). This demonstrates that these successive conversions were catalyzed by holobiont members and that this process starts as early as 12 h, suggesting that the similarly observed increase in SO_4_^2-^ was also derived from taurine. In *I. basta* explant incubations without externally added taurine (FSW + *I. basta*), NH_4_^+^ and NO_2_^−^ concentrations still greatly exceeded those in incubations without sponge explants and added taurine (FSW + taurine), providing additional support for the production of these compounds by *I. basta* holobiont members. Finally, HPLC analysis of taurine concentrations for these FSW incubations confirmed uptake of taurine by the *I. basta* holobiont, since incubations with *I. basta* explants and added taurine, exhibited concentrations (FSW + *I. basta* + taurine: 1.20 ± 0.05) significantly lower than in control incubations without *I. basta* explants and added taurine (FSW + taurine: 1.73 ± 0.02 mM taurine; Kruskal–Wallis, *p* < 0.05; Fig. S8).

Taken in totality, these incubation experiments demonstrate that the *I. basta* holobiont explants actively processed and incorporated taurine-derived carbon and nitrogen, while then dissimilating the resultant ammonia moiety to nitrite and oxidizing the sulfonate moiety to sulfate, from the onset through the course of the incubations. Together with the metaproteogenomic dataset, we conclude that ‘*Ca*. Taurinisymbion ianthellae’ is the holobiont member most likely responsible for this taurine incorporation and dissimilation, including the oxidation of the resultant sulfite to sulfate. Although the observed rates are potential net rates under specific incubation conditions, which will undoubtedly differ from in situ rates, as in all ex vivo experiments, the prompt incorporation and dissimilation of added exogenous taurine, suggests a N-limited sponge holobiont that is metabolically ready to access and transform this compound for anabolic as well as catabolic purposes. Recently, several laboratory and field-based studies have documented the exchange of sulfonates between marine bacteria and eukaryotic phytoplankton, with taurine (or its derivatives), always being one of the main sulfonates involved [32–34]. In co-culture, the coastal diatom *Pseudo-nitzschia multiseries* apparently provides a *Sulfitobacter* strain (SA11) with taurine in exchange for the plant hormone indole-3-acetic acid as well as ammonium [38]. Similarly, cross-feeding between nitrite oxidizers and ammonia oxidizers has been demonstrated wherein urea or cyanate is first processed by nitrite oxidizers [118, 119], thereby providing the cleaved ammonia for oxidation to nitrite. While marine Thaumarchaeota have been shown to directly utilize urea and cyanate [120] as well as taurine exuded by mesozooplankton [121], the mechanisms for the direct utilization of these dissolved organic compounds has yet to be elucidated and indirect utilization remains a confounding factor.

### A proposed model of an auxotrophy network between symbionts and the sponge host

In the *I. basta* metagenome, we found no indications for taurine biosynthesis by its microbial symbionts. Consequently, we believe it to be most likely that the taurine detected in the *I. basta* holobiont, is synthesized by the sponge, and after release, via export or sponge cell lysis, is utilized by ‘*Ca*. Taurinisymbion ianthellae’. In fact, the 5.5 µmol g^−1^ sponge wet weight taurine, we measured in *I. basta*, would correspond to ~6.6 mM concentrations within the sponge explants used for the incubations (under the assumption that 1.2 g sponge wet wt = 1 cm^−3^; [97]), and we further detected taurine concentrations of 1031.05 µM (SD ± 282.40 µM) within *I. basta* pore water. These taurine concentrations are on the high end of measured cytosolic concentrations in eukaryotic phytoplankton [34], which still does not account for further potential concentration effects in specialized cells, cell compartments, or in the mesohyl. We thus propose that endogenous taurine is either translocated by *I. basta* to ‘*Ca*. Taurinisymbion ianthellae’, as has been shown recently for DOM-derived carbon and nitrogen in a high microbial abundance (HMA) sponge [15], or is derived from host cell lysis, as a result of the rapid cell turnover characteristic of marine sponges [122, 123]. Nevertheless, it is possible that marine sponges themselves process taurine from the environment, as has been shown for bulk DOM mixtures [15, 17], instead of synthesizing it, and there remains the possibility that in our incubation experiments *I. basta* itself took up taurine and processed it. Even though our incubation experiments involved the addition of exogenous taurine, the almost immediate and continued production of the dissimilated and oxidized end products, ammonia, and sulfate, two processes for which, so far, only microbially mediated pathways have been demonstrated, suggests that the added taurine was processed primarily by the dominant gammaproteobacterial symbiont, ‘*Ca*. Taurinisymbion ianthellae’, and that it is metabolically ‘primed’ to process this compound. Alongside the incubation experimental results, as well as the measured taurine concentrations in *I. basta* bulk tissue and pore water, we therefore assume endogenous taurine supplementation by *I. basta* to be the most parsimonious route given the low taurine seawater concentrations [34, 121], competition with other microbes in seawater where taurine turnover rates are high [121], the ability of the sponge to be able to gain the taurine precursors cysteine and methionine from multiple sources (exogenous DOM and POM, as well as from its microbial symbionts), and the expression of the taurine degradation pathway by ‘*Ca*. Taurinisymbion ianthellae’ in vivo.

Taurine biosynthesis is typically considered to be a metazoan feature, but has not yet been demonstrated experimentally in sponges, nor has this capability been suggested in sequenced sponges. More recent discoveries have demonstrated bacterial and microalgal capabilities in synthesizing taurine [124, 125], and yet taurine biosynthesis in microalgae and bacteria is not universal and quite sporadic in distribution [34, 126]. Taurine biosynthesis pathways in mammalian and invertebrate tissues use cysteine as a precursor ([45] and references therein) and cysteine synthesis in metazoans requires methionine [127]. The methionine synthesis pathway, along with the biosynthesis pathways for several other essential amino acids, are generally lacking in metazoans [128–130], and thus these compounds are typically acquired via heterotrophy. While at least some sponges seem to possess the genomic repertoire for cysteine and methionine synthesis [131, 132], so far, no studies have confirmed their biosynthesis in radiolabeled amino acid precursor or feeding studies, whereas scleratinian corals can produce low amounts of methionine and perhaps even cysteine [132, 133]. ‘*Ca*. Taurinisymbion ianthellae’ encodes pathways for the synthesis of methionine and cysteine (Fig. S9), which are connected with the degradation of taurine and DMSP (Fig. 3), and also encodes several copies of EamA domain-containing proteins (IPR000620) and two copies of LeuE-type export proteins (IPR001123) (Fig. 3 and Table S5), with both families containing homologous representatives demonstrated to export cysteine [134] and methionine [135], respectively. Although further studies are needed to confirm whether marine sponges can indeed synthesize cysteine and methionine (as well as taurine), it is tempting to speculate that ‘*Ca*. Taurinisymbion ianthellae’ synthesizes and provides methionine and cysteine, either via active transport or symbiont cell lysis, to *I. basta*, and that the latter is used as a precursor for taurine biosynthesis. Cysteine or methionine auxotrophy in *I. basta* is not a precondition for this metabolic exchange to occur, as highly regulated kinetic switches may exist within the *I. basta* holobiont that is dependent on dynamic shifts in resource limitation and environmental stressors.

**Fig. 6:**
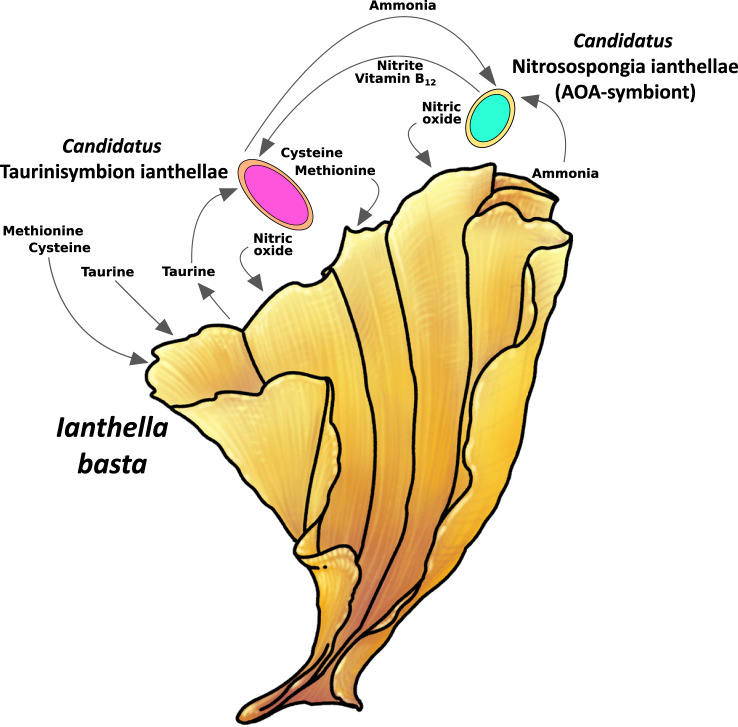
Conceptual scheme of tripartite metabolic interactions within the *Ianthella basta* holobiont. A proposed metabolic network between the dominant gammaproteobacterial symbiont, ‘*Candidatus* Taurinisymbion ianthellae’, the dominant ammonia-oxidizing thaumarchaeal symbiont, ‘*Candidatus* Nitrosospongia ianthellae’, the marine sponge host, *Ianthella basta*, and the surrounding environment.

Although the methionine synthase (MetH)-containing pathway for methionine biosynthesis in ‘*Ca*. Taurinisymbion ianthellae’ is vitamin B_12_ dependent (this organism also encodes a vitamin B_12_-independent pathway using DMSP as precursor, as well as the cobalamin-independent methionine synthase, MetE; Fig. 3, Fig S9), its MAG does not contain the genes required for synthesis of this vitamin, while it does encode a putative vitamin B_12_ transporter BtuCDF (Fig. 3 and Table S5). As the thaumarchaeal symbiont of *I. basta*, ‘*Ca*. Nitrosospongia ianthellae’, has the genomic repertoire for cobalamin (vitamin B_12_) biosynthesis [10], it is conceivable that it provides vitamin B_12_ for methionine biosynthesis in ‘*Ca*. Taurinisymbion ianthellae’ if DMSP is not available, while receiving ammonia dissimilated from taurine degradation. Nitrite formed by ammonia oxidation by ‘*Ca*. Nitrosospongia ianthellae’ can then serve under low oxygen concentrations as an electron acceptor in ‘*Ca*. Taurinisymbion ianthellae’. Furthermore, since nitric oxide has been shown to have an immediate contractile effect on sponges [136], and seems to be an ancient and key regulator in the marine sponge life cycle [137], the fact that ‘*Ca*. Taurinisymbion ianthellae’ encodes two denitrification genes — a nitrite reductase, *nirK* and a single subunit quinol-dependent nitric oxide reductase, *qNOR* [138] — that produces and consumes NO, suggests that this symbiont may be able to modulate host behavior. The frequent presence of an incomplete denitrification pathway along with the quinol-dependent *qNOR* — a nitrite reductase commonly found in pathogenic species [139] — in other sponge metagenomes [28], further points at the possibility that this is a widespread feature of microbial symbionts residing in marine sponges. Altogether, our data indicate a tightly connected metabolic network in the *I. basta* holobiont (Fig. 6), albeit in contrast to other selected sponges, no data are yet available for *I. basta* on the uptake of amino acids directly from the environment [16, 17] or from ingested free-living and symbiotic prokaryotes [140].

## Conclusions

In this study, we showed through a combination of metaproteogenomic analyses and incubation experiments combined with isotope labeling, that the dominant *I. basta* gammaproteobacterial symbiont, ‘*Ca*. Taurinisymbion ianthellae’, most likely uses endogenously occurring taurine that is potentially produced by the sponge holobiont, for assimilation and energy conservation. We experimentally demonstrated that the taurine dissimilation products, sulfate and ammonia, are secreted and that the latter compound is used for ammonia oxidation by the dominant thaumarchaeal symbiont — ‘*Ca*. Nitrosospongia ianthellae’. Taurine seems to be an important metabolite, not only in *I. basta*, but in many sponge holobiont systems, as indicated by the seeming ubiquity of taurine and taurine dissimilating genes within sponge microbiomes [25, 26, 35–37], and the appearance of a specific taurine-dissimilation/sulfite-oxidation pathway restricted mostly to symbiotic microorganisms [25, 26, 109]. Taurine also appears to be a central metabolite in several other metazoan holobiont systems such as in cnidarians, various bivalve species [44, 45, 141, 142], and in mammalian gut systems [39, 40]. Future research is needed to further disentangle the mechanisms of taurine synthesis via symbiont-derived or environmentally supplied precursors in marine sponge holobionts, to better understand the role of exogenous and endogenous taurine uptake, and the numerous roles taurine may play for the functioning of these symbioses.

## Supplementary information

Supplementary Information

Table S2

Table S3

Table S4

Table S5

## Data Availability

The metagenomic raw reads are available in the European Nucleotide Archive under BioProject accession number PRJEB9378. The MAG for ‘*Ca*. Taurinisymbion ianthellae’ was assembled from run ERR884062 and is available under the accession number ERS14904192. The proteomics raw data have been deposited to the ProteomeXchange Consortium via the PRIDE partner repository with the dataset identifier PXD012484 and 10.6019/PXD012484.
